# A message from the new Editor-in-Chief of the *Human Genome Variation*

**DOI:** 10.1038/s41439-020-0092-4

**Published:** 2020-03-19

**Authors:** Issei Imoto

**Affiliations:** Division of Molecular Genetics, Aichi Cancer Center Research Institute, Nagoya, Japan

It is with great pleasure that, from 1 January 2020, I have taken over the role of Editor-in-Chief from Professor Katsushi Tokunaga, who founded *Human Genome Variation (HGV)*, the second official journal of the Japan Society of Human Genetics, and worked diligently to increase its profile. For the last 6 years, the editorial committee has been successfully managed under his strong leadership. This open-access journal, launched in 2014, has attained a unique position in the human genetics/genomics research community. On behalf of the Editorial Board of the *HGV*, its authors and readers, I sincerely want to thank Professor Tokunaga for all his contributions to the journal.

During the years that I have been associated with *HGV* as a member of the Editorial Board, I have seen and experienced the success of this unique open-access, online-only journal. An innovative feature of the journal is a curated database of the underlying data from Data Reports, which is growing into an important resource for the human genetics/genomics community. The rapidly changing field of genomic technologies, especially next-generation sequencing and genome editing, are revealing more and more genetic variants responsible for or associated with various human diseases and traits. *HGV* aims to provide a coherent link between a published paper and the underlying data deposited in an accompanying database. This open-access database of variations and variability can be searched, filtered and variously used to further the progress of genomics research. Articles published in this journal are also fully open access, allowing for the widest dissemination, visibility, and impact of authors’ research. The intended audience for this journal is researchers, scientists, clinicians, genetic counsellors and those interested in human genetics/genomics, from all sectors and from around the world. We warmly welcome submissions from the scientific community. We assure authors submitting to the journal of efficient and respectful handling of their manuscripts and, in particular, of a shorter submission to decision time.

I am truly honored to assume the responsibility of the Editor-in-Chief of *HGV*, and also very proud to be working with an outstanding internationally diverse team of Associate Editors, members of the Editorial Board, reviewers, and the supporting staff in Springer Nature. As the new Editor-in-Chief, I will strive to continue to improve and enhance the quality and reputation of *HGV*. I welcome suggestions, discussions and thoughts from the readers, authors, reviewers, and editors to help me to understand and rectify any problems with the journal.

I am enthusiastic about this opportunity and look forward to working with you over the coming years.

